# Identification of deregulated lncRNAs in Alzheimer’s disease: an integrated gene co-expression network analysis of hippocampus and fusiform gyrus RNA-seq datasets

**DOI:** 10.3389/fnagi.2024.1437278

**Published:** 2024-07-17

**Authors:** Ermes Filomena, Ernesto Picardi, Apollonia Tullo, Graziano Pesole, Anna Maria D’Erchia

**Affiliations:** ^1^Department of Biosciences, Biotechnology and Environment, University of Bari Aldo Moro, Bari, Italy; ^2^Institute of Biomembranes, Bioenergetics and Molecular Biotechnologies, National Research Council, Bari, Italy

**Keywords:** Alzheimer’s disease, RNA-Seq, differentially expressed genes, lncRNAs, WGCNA

## Abstract

**Introduction:**

The deregulation of lncRNAs expression has been associated with neuronal damage in Alzheimer’s disease (AD), but how or whether they can influence its onset is still unknown. We investigated 2 RNA-seq datasets consisting, respectively, of the hippocampal and fusiform gyrus transcriptomic profile of AD patients, matched with non-demented controls.

**Methods:**

We performed a differential expression analysis, a gene correlation network analysis (WGCNA) and a pathway enrichment analysis of two RNA-seq datasets.

**Results:**

We found deregulated lncRNAs in common between hippocampus and fusiform gyrus and deregulated gene groups associated to functional pathways related to neurotransmission and memory consolidation. lncRNAs, co-expressed with known AD-related coding genes, were identified from the prioritized modules of both brain regions.

**Discussion:**

We found common deregulated lncRNAs in the AD hippocampus and fusiform gyrus, that could be considered common signatures of AD pathogenesis, providing an important source of information for understanding the molecular changes of AD.

## 1 Introduction

Alzheimer’s disease (AD) is a neurodegenerative disorder which, considering the growth of the global population coupled with the increasing life expectancy and the lack of effective therapies, is predicted to become one of the most high-impact health problems in the next few years. Two neuropathological hallmarks characterize the brain of AD patients: the accumulation of intraneuronal neurofibrillary tangles (NFTs) and the deposition of extracellular plaques, made up of beta-amyloid (Aβ) proteins, which are accompanied by synaptic loss, inflammatory and oxidative processes (Schapira et al., 2017).

Non coding RNAs (ncRNAs), as long non coding RNAs (lncRNAs), circular RNAs (circRNAs) and microRNA (miRNAs) are key regulators of many cellular processes and are known to be widely expressed in the brain where they play crucial roles in proliferation, survival, metabolism and differentiation of neuronal cells (Salta and De Strooper, 2017). Among ncRNAs, lncRNAs have received increasing attention as novel epigenetic regulators of gene expression at transcriptional and post-transcriptional levels (Nadhan et al., 2022). With the advancements in sequencing technology, transcriptomic studies progressively identify novel lncRNAs even if a comprehensive functional annotation is still lacking. It is estimated that about 40% of lncRNAs are specifically expressed in brain tissue, where they are involved in different brain physiological functions (Zimmer-Bensch, 2019; Srinivas et al., 2023). A deregulated expression of lncRNAs has been associated with neuronal injury in several neurodegenerative pathologies such as AD, Parkinson’s disease (PD), amyotrophic lateral sclerosis (ALS) and Huntington’s disease (HD), but how or whether they influence the onset of these diseases is still unclear (Srinivas et al., 2023). So far, the best documented lncRNA deregulation in AD concerns lncRNAs which are antisense transcripts of mRNAs derived from known AD-related genes, as *BACE1-AS, 51A, 17A* and *BC200*, which have been found to be directly involved in Aβ deposition, Tau iper-phosphorylation and neuroinflammation (Faghihi et al., 2008; Ciarlo et al., 2012; Ahmadi et al., 2020; Bagyinszky et al., 2020). Also, transcriptome analyses on post-mortem human brains have indicated that gene expression is significantly altered in AD patients (Cain et al., 2023), although the role of lncRNAs in the onset of the disease remains elusive. This evidence, as well as the possibility of their exploitation for new therapeutic strategies for AD, has progressively demanded a deeper investigation of the role of lncRNAs in AD (Balusu et al., 2023).

The present work aims at contributing to the current knowledge about the deregulation of lncRNAs in AD. For this purpose, we investigated 2 RNA-seq datasets: one derived from the hippocampus (Annese et al., 2018) and the other derived from the fusiform gyrus (Friedman et al., 2018) of AD patients. By using state of the art bioinformatic resources, a considerable number of differentially expressed (DE) genes was identified in these brain regions of AD patients, including lncRNAs. Comparing the DE genes between the two datasets, we found a set of 225 lncRNAs and 857 protein coding genes that were differentially expressed in both the brain regions. We performed a co-expression network analysis with WGCNA (weighted correlation network analysis) in order to infer the function of the DE lncRNAs, through a guilt-by-association view of transcriptomic expression, as the co-expression of protein coding and non-coding genes may suggest their involvement in the same pathway. We found some modules associated with neurotransmission and memory related pathways, such as CREB signaling in neurons and synaptic long term depression. By comparing the lncRNAs within the hippocampal and fusiform gyrus WGCNA prioritized modules, we identified common DE lncRNAs that could be considered common signatures of AD progression.

Our results thus could contribute to better defining the deregulated expression of AD brain and to explore new deregulated lncRNAs as potential targets for further investigation on molecular changes in AD pathogenesis.

## 2 Materials and methods

**Figure 1:**
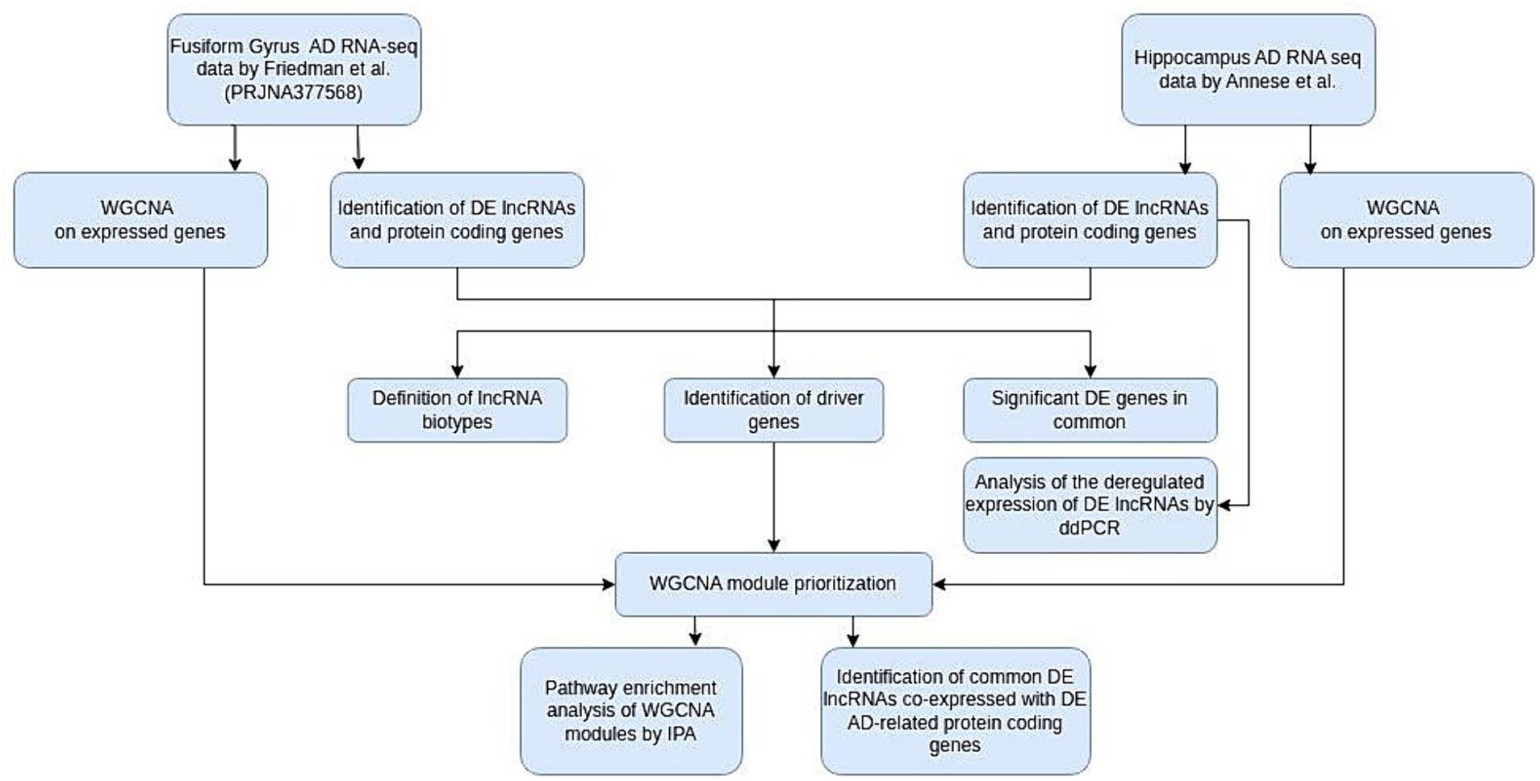
Flow chart of our study. AD, Alzheimer’s disease; WGCNA, weighted gene correlation network analysis; DE, differentially expressed; IPA, ingenuity pathway analysis; ddPCR, digital droplet PCR.

All experimental procedures performed are described in Figure 1.

### 2.1 RNA-seq datasets

The RNA-seq datasets used in this study were: (1) the Annese et al. dataset consisting of transcriptomic data from frozen post-mortem hippocampal samples derived from 6 AD donors and 6 healthy controls (Annese et al., 2018); (2) the Friedman et al. dataset (Bioproject PRJNA377568) (Friedman et al., 2018) downloaded via the dedicated ftp links from the Sequence Read Archive (SRA) database. The original 117 fusiform gyrus RNA-seq samples were filtered according to sex, age of death, ethnicity, patient Braak stage (V-VI) and RIN (RNA integrity number) value to match the stratification of the dataset by Annese et al. The final dataset analyzed comprised 28 samples, 14 AD subjects and 14 healthy controls. The sequencing output in the form of FASTQ files consisted in 166,295,065 reads on average per sample for the hippocampus dataset and 35,081,589 reads on average per sample for the fusiform gyrus dataset.

### 2.2 Data processing and RNA-seq data analysis

All computations were performed on machines running GNU+Linux (3.10.0–862.14.4.el7.x86_64), by using R (version 3.6.1) and Bash [4.2.46(2)-release x86_64-redhat-linux-gnu].

Data were analyzed according to the workflow reported in Supplementary Figure S1. All steps of the analysis dependent on genomic annotation employed the version 44 of GENCODE’s GTF and FASTA files, unless stated otherwise. The quality of the RNA-seq reads was preliminarily inspected with fastQC^1^ and MultiQC (Ewels et al., 2016). No trimming intervention was deemed necessary for the two datasets.

Both RNA-seq datasets were analyzed using different bioinformatic tools and updated annotations with respect to the original studies. Reads were summarized to genes via FeatureCounts (Liao et al., 2013) and GENCODE annotation and were aligned onto the human genome (GRCh38.p13) by means of STAR (Dobin et al., 2012), using the following options: (1) --chimFilter banGenomicN; (2) --outFilterMultimapNmax 1; (3) --alignSJoverhangMin 8; (4) --alignSJDBoverhangMin 1; (5) --outFilterMismatchNmax 2; (6) --outFilterScoreMinOverLread 0; (7) --outFilterMatchNminOverLread 0; (8) --outFilterMatchNmin 0; (9) --outFilterMismatchNoverLmax 0.04. For the hippocampal RNA-seq dataset, the sequence alignment with the reference genome uniquely mapped 83.2% of the ~2 billion input reads, namely, ~138 million reads per sample on average. For the fusiform gyrus RNA-seq dataset, the sequence alignment process uniquely mapped 91.9% of the ~982 million input reads, namely, ~32 million reads per sample on average.

DESeq2 (version 1.26.0) (Love et al., 2014) was used to perform the normalization of sequencing counts and the differential expression analysis between AD patients and relative controls. First, the summarized gene counts were normalized with DESeq2’s own method. This approach is commonly considered well suited to compare gene expression across samples and hence, to differential expression analyses. A preliminary gene expression filter was employed and genes, whose sum of normalized counts was less than 10 in half the samples of the datasets, were discarded. MDS and PCA analyses were performed, respectively, with the R functions “cmdscale” and “prcomp” to study the clustering behavior of samples; in particular, the Aitchison distance was adopted for MDS by using the dedicated parameter of the “cmdscale” function. Samples clustered according to their tissue in the MDS performed with the Aitchison distance and the regularized log-transformed (DESeq2 rlog function) counts. Control sample 5 (Supplementary Figure S2A) and control sample 4 (Supplementary Figure S2B) resulted as outliers in plots obtained with and without the rlog transformation, respectively. In the PCA biplot, HIP samples segregated according to their experimental condition only upon removal of control samples 4 and 5, which were ultimately considered outliers and removed from downstream analyses (Supplementary Figure S3). The PCA analysis performed for the fusiform gyrus samples showed that they did not cluster according to the condition (AD/CTL), however no outliers could be identified with ordination analyses nor sample removal improved the clustering (Supplementary Figure S4). The differential expression analysis was performed, after preparing the data as required by the DESeq2 package via a custom R script. Genes were considered as differentially expressed if Benjamini-Hochberg adjusted *p*-value (padj) resulted inferior to 0.05.

### 2.3 Differentially expressed lncRNAs biotype definition

By parsing the GENCODE annotation with a custom R script, the following biotypes were used to classify the DE lncRNAs of the two datasets: (i) intergenic: the lncRNA that does not overlap any protein coding gene; (ii) antisense: the lncRNA that overlaps a protein coding locus on the opposite strand; (iii) sense overlapping: the lncRNA that has a transcript overlapping a coding gene’s exon on the same strand; (iv) sense intronic: the lncRNA that falls in introns of a coding gene and do not overlap any exon. More specifically, the genomic coordinates of the starting and ending points of annotated genes were compared to those of the DE lncRNAs. Boolean vectors (i.e., lists of TRUE and FALSE values) obtained from the comparisons were logically chained through AND/OR operators to verify the overlap events and their nature. An analogous mechanism was applied to elucidate the intronic or exonic nature of the overlap events.

### 2.4 Weighted gene co-expression network analysis

The WGCNA R package (Langfelder and Horvath, 2008) (version 1.69) has been employed to run a weighted gene co-expression network analysis of the coding and non-coding genes obtained in the differential expression analysis of the two RNA-seq datasets. The pickSoftThreshold function was used with the ‘networkType’ parameter set to ‘signed’ to produce the data for the plots data necessary to choose the soft thresholding power β of the correlation function necessary to build an adjacency matrix based on gene expression (Supplementary Figure S5). Raising the absolute value of the correlation between genes to the soft thresholding β power allows to underline disparity between correlations in the adjacency matrix. β should be a good compromise between the scale free topology model fit and the consequent network mean connectivity. For the hippocampal dataset, we chose soft thresholding power *β* = 15 (Supplementary Figures S5A,B), while for the fusiform gyrus dataset we chose *β* = 18 (Supplementary Figures S5C,D). The correlation function chosen was run combinatorially between the expression data of all genes of interest to generate an adjacency matrix. A topological overlap matrix (TOM) was obtained from the adjacency matrix and finally, a 1-TOM dissimilarity matrix was calculated (Langfelder and Horvath, 2008). The gene distances of the dissimilarity matrix calculated in the previous step of the workflow were used to build the dendrograms with the WGCNA function plotDendroAndColors. The hierarchical clustering analysis was performed with the flashClust R package (version 1.1.2) and with the option ‘method = average’. The dynamic tree cutting procedure was applied with the following main parameters: ‘minClusterSize = 30’ and ‘deepSplit = 2’. Close WGCNA modules were merged with the ‘cutHeight’ set to 0.12. Gene networks representations were obtained with the igraph R package (version 1.3.2). The data of the two weighted correlation networks were prepared with the “exportNetworkToCytoscape” function of the WGCNA R package, then, the network graphs were generated with the fruchterman-reingold layout and they were finally pruned with the threshold option so that only edges whose weight resulted bigger than or equal to 0.385 for the hippocampus dataset and to 0.27 for the fusiform gyrus were retained in the final representation (These thresholds were determined empirically). WGCNA modules were prioritized according to: (i) their number of differentially expressed lncRNAs; (ii) their number of differentially expressed driver lncRNAs. Driver genes were considered as such within a module when showing a Pearson’s correlation |*r*| > 0.8 to the module’s eigenvector and to the experimental condition of interest, namely, AD. For the fusiform gyrus, the |*r*| threshold for correlation of genes to the trait of interest was lowered to 0.6 as no genes passed the more stringent filter (|*r*| = 0.8 threshold).

### 2.5 Pathway enrichment analysis

The functional pathways associated with genes in WGCNA prioritized modules were investigated with Ingenuity Pathway Analysis IPA® (Ingenuity Systems, QIAGEN, Redwood City, CA). IPA parameters were kept to their standard values except for species settings (in the species tab, only the *Homo sapiens* checkbox was considered) and miRNA settings (the box for high confidence predicted miRNAs was checked). Possible connections with AD were sought in the ‘diseases and functions’ and ‘canonical pathways’ tabs of the analysis report produced by IPA whose content was exported through the dedicated functions. Gene modules were considered to be associated with a canonical pathway if the Fisher’s exact test performed by IPA was significant (*p*-value <0.05).

### 2.6 RNA-seq analysis validation by ddPCR

To validate RNA-seq data, primer pairs for *MAP4K3-DT, MEG9, MEG8, PCA3, HAR1A*, *NECTIN3-AS1, STARD4-AS* lncRNAs were designed by using an *ad-hoc* developed pipeline (Supplementary Table S1). Total RNA from frozen post-mortem hippocampus samples used in the Annese et al. work (Annese et al., 2018) was used for the lncRNAs expression validations. Samples were processed in accordance to Annese et al. study (Annese et al., 2018), which was approved by the Institutional Review Board of the Institute of Biomembranes, Bioenergetics and Molecular Biotechnologies, National Research Council. 1.5 μg of RNA were used in the reverse transcription reaction, using the iScript™ Advanced cDNA Synthesis Kit (Bio-Rad Laboratories Ltd., Berkeley, California, USA), according to the manufacturer’s instructions. The droplet digital polymerase chain reaction (ddPCR) (Bio-Rad) was chosen for the quantification analysis. All ddPCR reactions were carried out in a final volume of 22 μL, using the QX200™ ddPCR™ EvaGreen Supermix, and were prepared according to the manufacturer’s instructions. The amount of the cDNA template was determined empirically for the different targets, as reported: 1 μL of diluted cDNA (1:4) for *MAP4K3-DT, MEG9, MEG8, PCA3, HAR1A*; 2 μL of undiluted cDNA for *NECTIN3-AS1, STARD4-AS;* 1 μL of diluted cDNA (1:100) for *GAPDH*. Primer concentration in reaction was 200 nM for all targets except for *PCA3* (150 nM). Each RNA sample was analyzed in duplicate. For each experiment, a negative control (No Template Control, NTC) was used. After droplet generation with the QX200 Droplet Generator (Bio-Rad), droplets were transferred into a 96-well plate that was sealed for PCR. The thermal cycling conditions were set as recommended by the manufacturer, except for the annealing/extension temperature and the number of the cycles that were adapted to each target (Supplementary Table S2). Absolute quantification was performed using the QuantaSoft version 7.4.1 software (Bio-Rad) and the negative/positive thresholds were set manually. ddPCR reactions were considered positive if characterized by a number of events >10,000, according to the QX200™ reader automatic evaluation. For each sample, results were expressed as the means of the lncRNA copies/μL of PCR replicates, normalized by the means of corresponding *GAPDH* copies/μL. Statistical significance was evaluated with a two tailed Mann–Whitney U test that was performed with the wilcox.test R function.

### 2.7 Statistical analyses

The RNA-seq sample cohorts analyzed were homogenous in terms of sex, ethnicity, age at death, quality of the input RNA and Braak stage for the patients, therefore the differential expression analysis was performed by applying a Wald test for each dataset via the dedicated DESeq2 functions considering only the condition in the formula. For ddPCR analysis, statistical significance was assessed by using and a two tailed Mann–Whitney *U* test and results were expressed as the means of lncRNA copies/μL, normalized with the means of *GAPDH* copies/μL for each sample.

## 3 Results

### 3.1 Identification of differentially expressed genes in the hippocampus and fusiform gyrus in AD

In this study we re-analyzed the data produced in two RNA-seq experiments performed on post-mortem AD brain tissues. The first was produced by Annese et al. in 2018 and consisted of the transcriptomic profiles of the hippocampal CA1 region of six patients affected by late-onset AD and six cognitively unimpaired controls (Annese et al., 2018). The second dataset was produced by Friedman et al. in 2018 and originally consisted of 117 total RNA-seq samples from the fusiform gyrus (84 AD, 33 controls) (Friedman et al., 2018). From this dataset, we chose samples according to the same sampling criteria of the hippocampal cohort, obtaining a final cohort comprising 14 controls and 14 AD fusiform gyrus samples (Supplementary Table S3).

By using DESeq2, we identified 3,297 protein coding genes and 1,180 lncRNAs as differentially expressed (DE) genes between hippocampal AD samples and controls (padj <0.05). In particular, 567 lncRNAs were found down-regulated and 613 up-regulated; among protein coding genes, 1,416 genes were found down-regulated and 1,881 up-regulated (Table 1 and Supplementary Table S4). For the fusiform gyrus RNA-seq dataset, DESeq2 identified 3,728 DE genes (padj <0.05), of which 2,871 were protein coding genes and 857 were lncRNAs. In particular, among protein coding genes, 1,324 were down-regulated and 1,547 were found to be up-regulated while, of the DE lncRNAs, 382 were found to be down-regulated and 475 were up-regulated (Table 1 and Supplementary Table S4).

**Table 1:** Number of deregulated genes (lncRNAs and protein coding genes) identified in the hippocampus and fusiform gyrus of AD patients and in common between the two datasets.

	**Hippocampus**		**Fusiform gyrus**		**Common DE genes**	
**Genes**	**lncRNAs**	**Protein coding RNAs**	**lncRNAs**	**Protein coding RNAs**	**lncRNAs**	**Protein coding RNAs**
Analyzed in total	8,431	17,362	6,660	16,724	5,787	16,553
Deregulated	1,180	3,297	857	2,871	225	857
Downregulated	567	1,416	382	1,324	92	293
Upregulated	613	1881	475	1,547	133	564

Genes with padj <0.05 were considered deregulated.

Thanks to the progressive improvement of lncRNA annotation, for both RNA-seq datasets, we identified more DE lncRNAs than the previous analyses (1,180 significant DE lncRNAs versus 47 for the hippocampus and 857 versus 65 for the fusiform gyrus).

Comparing the DE genes between the two datasets, we found a total of 1,082 DE genes (225 lncRNAs and 857 protein coding genes) in common (Table 1), the majority of which had the same expression pattern, while 39 genes (7 lncRNAs and 32 protein coding genes) showed an opposite expression behavior in the two brain regions.

### 3.2 DE lncRNAs biotype characterization

As one of the aims of this work was the characterization of DE lncRNAs in AD, firstly we analyzed the genomic neighborhood of the DE lncRNAs identified in AD hippocampus and fusiform gyrus, using a custom R script to retrieve information by processing the genomic coordinates reported by GENCODE’s annotation. In Table 2, the number of the DE lncRNAs for each biotype class is reported. Although the attribution of biotype is susceptible to changes due to annotation and considering that some lncRNAs fell into multiple biotype categories according to their topology (as *FLNC-AS1*, which is both sense overlapping to *KCP* gene and antisense to *FLNC* gene), our analysis reported that the greatest number of DE lncRNAs belongs to the sense-overlapping biotype in both brain districts, followed to the antisense biotype for the hippocampus. For the fusiform gyrus, an almost equal number of antisense and intergenic lncRNAs was identified.

**Table 2:** Number of differentially expressed lncRNAs identified in the hippocampus and in the fusiform gyrus of AD patients, grouped according to their biotype.

lncRNA biotype	Hippocampus	Fusiform Gyrus
Antisense	1,191	1,090
Intergenic	588	357
Sense intronic	112	59
Sense overlapping	3,393	2,926

### 3.3 Experimental validation of the differential expressed lncRNAs in AD hippocampus

**Figure 2:**
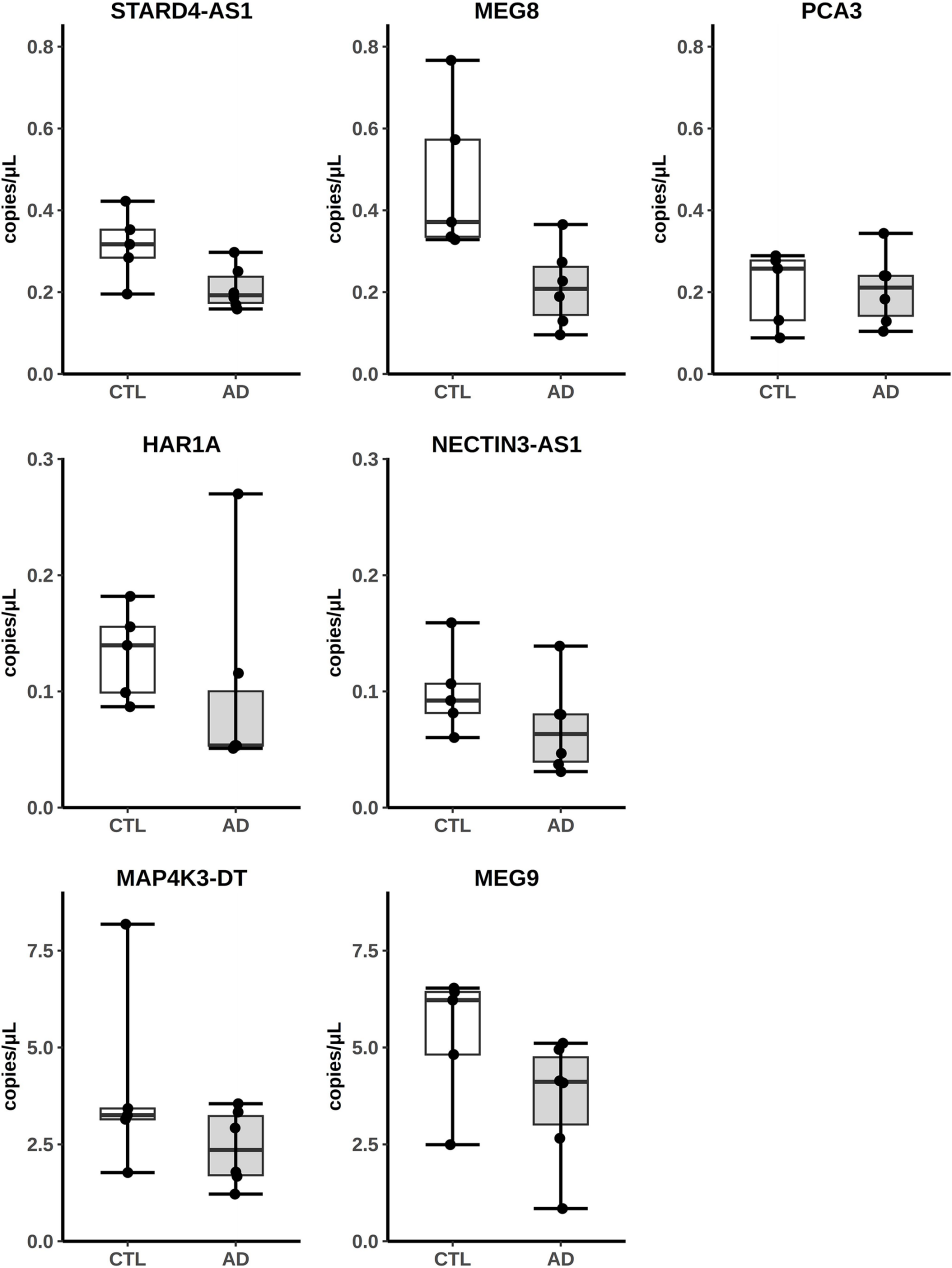
Analysis of lncRNAs expression from AD hippocampus RNA-seq data by ddPCR. Results are expressed as copies/μL of reaction mix values, normalized with respect to GAPDH copies/μL.

The expression of some hippocampal DE lncRNAs was validated by absolute quantitation with Digital Droplet PCR (ddPCR) on five controls (four original controls plus a new one) and six AD patients (five original AD subjects plus a new patient). The new samples were chosen to fit the selection criteria adopted for the original cohort used in the RNA-seq analysis (Supplementary Table S3). Seven downregulated lncRNAs, *MAP4K3-DT, MEG9, MEG8, PCA3, HAR1A, NECTIN3-AS1* and *STARD4-AS*, were selected for validation, as they appeared among the most differentially expressed and their nomenclature was approved by the HUGO Gene Naming Committee (HGNC). As shown in Figure 2, for all lncRNAs analyzed, the downregulated expression was confirmed by ddPCR. In fact, for each lncRNA, the obtained values of copies/μL of reaction mix, normalized by dividing them with respect to the GAPDH copies/μL, were lower in AD samples compared to controls, although we did not observe a statistically significant difference between the two groups.

### 3.4 Correlation network analysis of lncRNAs in AD hippocampus and fusiform gyrus

The Weighted Gene Correlation Network Analysis (WGCNA) tool (Langfelder and Horvath, 2008) was employed to analyze the co-expression network of coding genes and lncRNAs in AD samples. By choosing the appropriate soft thresholding power β, the TOM and the 1-TOM dissimilarity matrices were obtained. The hierarchical clustering of the dissimilarity matrix data generated the dendrograms shown in Supplementary Figures S6A,B for the hippocampus and the fusiform gyrus datasets, respectively. Each branch of the dendrograms harbors one of the genes (lncRNA and protein coding genes) considered. After the dynamic module merging procedure, 56 modules were identified for the hippocampus dataset and 52 for the fusiform gyrus dataset. For both datasets, the correlation networks obtained, the hierarchical dendrograms as well as the modules before and after the dynamic merging process are represented in Supplementary Figures S6C,D. Next, modules were prioritized to perform a pathway enrichment analysis in order to identify the physiological function and/or the biological pathway common to multiple protein coding genes and to the co-expressed lncRNAs present in a module. Two criteria were adopted to prioritize the modules: (i) the number of DE lncRNAs present in the module; (ii) the number of DE “driver” lncRNAs present in the module. Driver genes are key genes that may influence the expression or function of other genes or may be causal factors for a trait of interest. The top 10 modules comprising the largest number of significant DE lncRNAs are listed in Supplementary Table S5, and the top 10 modules comprising the largest number of DE “driver” lncRNAs are listed in Supplementary Table S6, for both datasets. Comparing the two lists for each dataset and considering the number of total and driver DE lncRNAs present in the modules, the top four ranking modules resulted “purple,” “lavenderblush3,” “grey60” and “brown” for the hippocampus dataset and “lavenderblush3,” “brown,” “turquoise” and “darkturquoise” for the fusiform gyrus dataset. Although some modules have the same name (e.g., “lavenderblush3”) for both hippocampal and fusiform gyrus datasets, these modules are independent clusters of genes, as names to the modules were automatically assigned by WGCNA.

### 3.5 Pathway enrichment analysis of lncRNAs present in prioritized WGCNA modules

The “purple,” “lavenderblush3,” “grey60” and “brown” selected modules of the hippocampal dataset and the “brown,” “turquoise,” “lavenderblush3” and “darkturquoise” for the fusiform gyrus dataset were subjected to a pathway enrichment analysis by using Ingenuity Pathway Analysis (IPA). Gene modules were considered to be associated with a canonical pathway if the pathway enrichment test indicated a significant enrichment within the module, with a *p*-value <0.05.

Regarding the hippocampal modules, the “purple” and “grey60” modules were found to be associated with the “CREB signaling in neurons” and the “synaptic long-term depression” pathways, as they included genes, like *GRIA1, DRD5, PRKCG, CACNA1E, CACNG4, PLCZ1, ADGRG4* and *GPR83,* that are neurotransmission-related (Supplementary Table S7). The first pathway is involved in the process of consolidating a new memory and the dynamic complexity of information processing within neuronal networks, which is greatly increased by activity-dependent changes in gene-expression within individual neurons (Silva et al., 1998). The “synaptic long term depression” pathway is described as a cellular model for information storage and synaptic plasticity (Ito, 2001). The brown module was found to be associated with the “synaptogenesis signaling,” “synaptic long-term potentiation,” “SNARE signaling” and “CREB signaling in neurons” pathways, as it included genes like *CALM1, GRIA2, GRIN2B, EPHA4, STXBP1, PPP3R1,* and *WASF1*. The “lavenderblush3” module was associated to GABA receptor and calcium signaling pathways although the *p*-value of the enrichment test was not significant; interestingly, this module was associated to the *TP53* signaling pathway, since *TP63* and *TP73* were downregulated in our analysis (Supplementary Table S7).

Regarding the fusiform gyrus modules, the “brown” module, which included genes like *CDH7, CDH18, PAK1, PPP1R14C* and *PRKCE,* was found to be associated with “synaptogenesis signaling” and “synaptic long-term potentiation” pathways. The “lavenderblush3” module was related to the downregulation of the “CREB signaling in neurons” pathway as it included *CAMK4, CAMK2D* and *FZD3* genes. Finally, the “darkturquoise” module was found associated with the “synaptic long term potentiation” pathway for the presence of *CREBBP*, *EP300* and *RAF1* genes, while the “turquoise” module was not found associated with AD or neurotransmission-related pathways (Supplementary Table S8).

Considering the co-expression analysis performed, it may be inferred that lncRNAs clustered in these modules could be related to AD as well.

As the prioritized modules from both brain regions correlated to common canonical pathways, we compared these modules to select the common DE coding and non-coding genes between AD hippocampus and fusiform gyrus. As shown in Table 3, all hippocampal modules share DE genes with the fusiform gyrus modules and, in particular, the “brown” and “purple” modules share a higher number of DE genes with the “brown” and “lavenderblush3” modules of the fusiform gyrus.

**Table 3:** Significant differentially expressed genes (lncRNAs and protein coding genes) in common between the hippocampus (Hip) and fusiform gyrus (Fg) prioritized modules.

Hip brown module vs. Fg brown module	Hip purple module vs. Fg brown module	Hip grey60 module vs. Fg brown module	Hip lavenderblush3 module vs. Fg brown module
CCR6 TSPYL2 CADPS2 SH2D5 UROS PCSK2 DGAT2 DRP2 GABRA3 MAGEE1 ADD2 NELL2 PRICKLE1 SGIP1 DCTN1-AS1* ENSG00000272121* RFPL1S* FLRT2-AS1* ENSG00000283538* ENSG00000260163* ENSG00000274718*	TARBP1 PTK2B SLITRK3 PCDH8 CCDC171 COG1 DRD5 ADAT2 LINC01962* ENSG00000261026* ENSG00000287805* LINC00839*	CFAP92 RNF165 MAP4K3-DT*	CHGB LINC00504*

**Hip brown module *vs* Fg lavenderblush3 module**	**Hip purple module *vs* Fg lavenderblush3 module**	**Hip grey60 module *vs* Fg lavenderblush3 module**	**Hip lavenderblus3 module *vs* Fg lavenderblush3 module**
ARHGEF28 TAFA1 MRTFB NDST3 ATP6V1H PLK2 ARHGEF9 ERICH3 ST8SIA3 NWD2 CAMK4 ENC1 NELL1 PCDH7 LRATD1 EXOC6 ATRNL1 GRIK2 CRACDL ATP2B1-AS1* LINC00390* ENSG00000278727*	MTMR1 CALB1 ST6GALNAC5 GRHL1 TMEM241 NEUROD1 EGR3 EXT1 GJD3 ZDHHC23 SLC22A25 INHBA-AS1* ENSG00000230393* LETR1* ENSG00000286389*	GPR83 ENSG00000285652* ENSG00000287900* ENSG00000288020*	TTLL1 SLC30A3 DNAJC5G ENSG00000286888*

**Hip purple module *vs* Fg darkurquoise module**	**Hip grey60 module *vs* Fg darkurquoise module**	**Hip lavenderblus3 module *vs* Fg darkurquoise module**	**Hip brown module *vs* Fg darkurquoise module**
ENSG00000226149*	–	–	–

**Hip purple module *vs* Fg turquoise module**	**Hip grey60 module *vs* Fg turquoise module**	**Hip lavenderblus3 module *vs* Fg turquoise module**	**Hip brown module *vs* Fg turquoise module**
PROC	–	ENO4	–

*lncRNA.

A functional enrichment analysis was performed by IPA on these common DE genes that highlighted the presence of six protein coding genes which are known to be related to AD: (i) *GABRA3*, encoding for a subunit of the GABA receptor, was found downregulated in the AD middle temporal gyrus (Govindpani et al., 2020); (ii) *CALB1*, encoding for a calcium binding protein, was found downregulated in AD hippocampal granular layer (Palop et al., 2003); (iii) *SLC30A3*, also called *ZNT3,* encodes for a synaptic vesicular Zn^2+^ transporter, whose loss was associated with synaptic and memory deficits of AD (Adlard et al., 2010); (iv) *PLK2* encodes for a kinase found upregulated in human AD cortex (Mbefo et al., 2010); (v) *NDST3*, encodes for a strong regulator of the autophagy-lysosomal pathway whose dysregulation, associated with proteostatic imbalance, is a hallmark of neurodegenerative diseases (Tang et al., 2021); (vi) *DRD5,* encodes for the dopamine receptor 5 and an antagonist molecule of this receptor, called Olanzapine, is in phase 4 of clinical trial for the treatment of AD (Mühlbauer et al., 2023).

To characterize the lncRNAs co-expressed with these six AD related genes in hippocampus and fusiform gyrus, we considered the 1-TOM dissimilarity matrices obtained during the clustering analysis and we obtained a list of DE lncRNAs correlated to these protein coding genes because they were part of the same WGCNA module (Tables 4, 5). By comparing the results of these analyses, we identified 6 DE lncRNAs that are correlated to the same AD related genes in both hippocampus and fusiform gyrus (Table 6). Three of these lncRNAs are antisense (*RFPL1S*, *DCTN1*-*AS*, *ATP2B1*-*AS*), one is a sense overlapping RNA (*LINC00390*) and 2 lncRNAs belong to the intergenic class (*ENSG00000274718* and *ENSG00000278727*).

**Table 4:** lncRNAs that are co-expressed with AD-related protein coding genes, according to the 1-TOM dissimilarity matrix in hippocampus (Hip) prioritized modules.

Gene	Hip module	Correlated lncRNAs
GABRA3	Brown	ENSG00000259985, RFPL1S, ENSG00000230051, ENSG00000271755, ATP2B1-AS1, LINC02144, NUP50-DT, ENSG00000271882, CNIH3-AS2, ENSG00000261292, DHX9-AS1, PLPPR5-AS1, DLX6-AS1, RNF32-DT, LINC02023, ENSG00000287527, TRIM7-AS2, ENSG00000266335, ENSG00000272163, RAPGEF4-AS1, PARTICL, LINC00239, ENSG00000284707, HAR1A, ENSG00000274718, ENSG00000254921, INKA2-AS1, ENSG00000286391, LINC00390, KIF18B-DT, ENSG00000283743, ENSG00000253596, UNC5C-AS1, LINC02440, SLC26A4-AS1, DCTN1-AS1, ENSG00000286736, ENSG00000260196, ENSG00000261135, ENSG00000286918, ENSG00000260482, ARMCX5-GPRASP2, ENSG00000260464, NA, REPIN1-AS1, LINC02283, ENSG00000270883, ENSG00000261654, ENSG00000286129, LINC01208
CALB1	Purple	ENSG00000260328, KCNK4-TEX40, LETR1, ENSG00000251680, ENSG00000256596, ENSG00000286282, ENSG00000261026, STARD4-AS1, ENSG00000253121, PFN2-AS1, LINC01494, ENSG00000249150, LINC00457, ENSG00000286230, LINC01621, LINC00839, TMCC1-DT, ERICH6-AS1, FGGY-DT, ENSG00000272247, LINC00571, ENSG00000287867, LINC01119, ENSG00000257194, ENSG00000245768, ENSG00000235450, CFAP20DC-DT, TDRKH-AS1, FLJ20021, MIR130AHG, BHLHE22-AS1, ENSG00000227606, LINC01547, LINC00184, ENSG00000286111, LINC03040, MEF2C-AS2, ENSG00000285679, ENSG00000228151, ENSG00000269107, LINC00943, NA, ENSG00000271727, NA, ENSG00000259199, ENSG00000236958, ENSG00000253355, NECTIN3-AS1, ENSG00000287468, ENSG00000228222
SLC30A3	Lavenderblush3	ENSG00000251187, MGC4859, ENSG00000280145, ENSG00000287832, LINC01267, ENSG00000228162, LINC01571, ENSG00000259628, ENSG00000288015, ENSG00000247311, CYP4A22-AS1, LINC02688, ENSG00000259222, UCHL1-DT, ENSG00000145075, ENSG00000236106, LINC01765, ACBD3-AS1, LINC01014, CNTN4-AS1, ENSG00000270265, LINC00907, USP3-AS1, CCNO-DT, IGFBP7-AS1, ENSG00000285930, KIF23-AS1, ENSG00000249621, LINC02742, LINC00504, ENSG00000258752, ENSG00000286777, LINC02525, ENSG00000283383, ENSG00000286472, EWSAT1, ENSG00000249631, LINC03053, ENSG00000277010, FHAD1-AS1, LINC02838, ENSG00000287427, ENOX1-AS2, ENSG00000288040, LINC02133, TSBP1-AS1, ENSG00000276842, ENSG00000227712, ENSG00000253796, SPATA42
PLK2	Brown	ENSG00000259985, RFPL1S, LINC02023, ATP2B1-AS1, CNIH3-AS2, LINC00390, ENSG00000287527, ENSG00000271882, ENSG00000274718, ENSG00000266335, ENSG00000254921, INKA2-AS1, ENSG00000230051, NUP50-DT, ENSG00000261292, PLPPR5-AS1, RNF32-DT, HAR1A, ENSG00000286391, RAPGEF4-AS1, DLX6-AS1, PARTICL, ENSG00000271755, ENSG00000229976, LINC02440, LINC00239, TRIM7-AS2, DCTN1-AS1, LINC02144, DHX9-AS1, LINC01829, ENSG00000283743, ENSG00000253596, ENSG00000287816, MEG8, ENSG00000260482, ENSG00000270883, NA, KIF18B-DT, ENSG00000254040, ENSG00000286736, REPIN1-AS1, ENSG00000272163, ENSG00000286675, NA, CD101-AS1, ENSG00000278727, MACROD2-IT1, LINC02389, MKNK1-AS1
NDST3	Brown	RFPL1S, LINC02023, DCTN1-AS1, ATP2B1-AS1, ENSG00000259985, ENSG00000287068, CNIH3-AS2, MEG8, ENSG00000286675, LINC02389, LINC02440, INKA2-AS1, ENSG00000229976, NA, ENSG00000260920, ENSG00000272121, MKNK1-AS1, ENSG00000272944, ENSG00000278727, ENSG00000287527, ENSG00000270883, ENSG00000272420, ADAMTS19-AS1, SNHG14, ENSG00000255910, ENSG00000261292, ENSG00000255448, ENSG00000227681, ENSG00000266335, SNAP25-AS1, NUP50-DT, ENSG00000287976, ENSG00000249738, RNF32-DT, CD101-AS1, MYCNOS, ENSG00000287887, LINC01208, ENSG00000288062, LINC02740, ENSG00000261553, ENSG00000286716, LINC01123, LINC00621, ENSG00000260163, ZNF567-DT, MYG1-AS1, LINC01829, NA, ENSG00000268288
DRD5	Purple	ACTR3-AS1, LETR1, ENSG00000229588, ST20-AS1, LINC01501, ENSG00000272106, LINC00839, ENSG00000283445, KCNK4-TEX40, ENSG00000287844, ENSG00000235450, ENSG00000259560, ENSG00000286066, LINC01213, ENSG00000286282, ENSG00000236823, BHLHE22-AS1, ENSG00000256596, ENSG00000260328, CORO1A-AS1, ENSG00000246308, ENSG00000226149, ENSG00000271727, FLJ20021, LINC01119, ENSG00000231918, ENSG00000286867, LINC00943, ENSG00000287468, ENSG00000236744, LINC01358, ENSG00000269107, TRIM36-IT1, ENSG00000228151, INHBA-AS1, ENSG00000286389, NA, ENSG00000261411, ENSG00000286719, ENSG00000271901, ENSG00000287477, ENSG00000253507, ENSG00000287204, LINC02802, ENSG00000287255, STARD4-AS1, LINC01879, ENSG00000227598, ENSG00000285898, AFF2-IT1

**Table 5:** lncRNAs that are co-expressed with AD-related protein coding genes, according to the 1-TOM dissimilarity matrix, in prioritized fusiform gyrus (Fg) modules.

Gene	Fg module	Correlated lncRNAs
GABRA3	Brown	FLRT2-AS1, RFPL1S, COPG2IT1, ENSG00000251095, ENSG00000258768, ENSG00000260163, ENSG00000274718, ENSG00000258945, ENSG00000255202, ENSG00000267396, ENSG00000283183, MSC-AS1, ENSG00000258931, TSC22D1-AS1, ENSG00000257522, ENSG00000197332, ENSG00000259678, LINC01182, ENSG00000283538, ENSG00000286282, ENSG00000286971, ENSG00000272944, DCTN1-AS1, ARMCX5-GPRASP2, ENSG00000287241, ANKRD34C-AS1, SPNS2-AS1, ENSG00000281160, LINC02857, MAP4K3-DT, LIN28B-AS1, LINC01007, ENSG00000288062, ENSG00000255910, DGCR5, RAPGEF4-AS1, ENSG00000287315, ZIM2-AS1, ENSG00000258035, NA, ENSG00000286125, ENSG00000240086, LINC02035, ENSG00000251680, ENSG00000248559, ENSG00000287769, LINC01963, NA, LUARIS, ENSG00000266573
CALB1	Lavenderblush3	ENSG00000273275, LY86-AS1, SLC26A4-AS1, ENSG00000261037, ENSG00000278727, ENSG00000236064, MAL2-AS1, LINC01332, ENSG00000229618, ENSG00000260412, ENSG00000285634, LETR1, ENSG00000256538, ENSG00000287038, ENSG00000249684, ENSG00000223944, LINC01331, LINC01476, INHBA-AS1, MIR4500HG, ENSG00000255595, ATP2B1-AS1, LINC00507, ENSG00000233928, NA, CFAP20DC-AS1, ENSG00000287018, LINC02009, LINC01885, LINC02346, ENSG00000285582, ENSG00000255087, ENSG00000253452, ENSG00000254664, CYP1B1-AS1, ENSG00000285572, ENSG00000284428, ENSG00000286771, ACAP2-IT1, ENSG00000253762, ENSG00000286888, LINC01250, ENSG00000278911, ENSG00000248837, ENSG00000240291, ENSG00000262267, ENSG00000286198, ENSG00000249453, ENSG00000255372, MAP3K4-AS1
SLC30A3	Lavenderblush3	LY86-AS1, ENSG00000236064, SLC26A4-AS1, ENSG00000273275, LINC02885, ENSG00000260412, LETR1, ENSG00000253762, CFAP20DC-AS1, LINC01476, MIR4500HG, ENSG00000251600, ENSG00000287690, LINC01250, LINC02346, ENSG00000253452, ENSG00000284703, ENSG00000255595, ATP2B1-AS1, ENSG00000285634, INHBA-AS1, LINC00343, ENSG00000255087, LINC03026, ENSG00000223944, LINC00390, LINC01332, LINC01616, PYDC2-AS1, ENSG00000253553, ENSG00000248837, ENSG00000286961, LINC00642, LINC01331, ENSG00000283403, ENSG00000278727, THSD4-AS1, CYP4F26P, ENSG00000233928, MIR137HG, ENSG00000229618, ENSG00000285966, ENSG00000283294, ENSG00000224404, ENSG00000286934, ENSG00000287271, MEG3, NA, ENSG00000261037, SNAP25-AS1
PLK2	Lavenderblush3	LETR1, LY86-AS1, CFAP20DC-AS1, SLC26A4-AS1, LINC00390, LINC02346, ATP2B1-AS1, ENSG00000260412, MIR137HG, ENSG00000253553, MIR4500HG, ENSG00000236064, ENSG00000233928, LINC01616, ENSG00000285634, ENSG00000278727, LINC01885, ENSG00000273275, ENSG00000287900, ENSG00000287439, ENSG00000285582, ENSG00000286720, ENSG00000224404, ENSG00000287690, LINC03026, LINC00642, LINC01618, LINC00488, ENSG00000286447, LINC01476, INHBA-AS1, ENSG00000285572, SNAP25-AS1, ENSG00000253452, ENSG00000251600, NA, THSD4-AS1, MAL2-AS1, MAP3K4-AS1, MEG3, ENSG00000284703, PYDC2-AS1, LINC02885, ENSG00000255595, ENSG00000285966, LINC01250, ENSG00000248837, ENSG00000286386, ENSG00000255087, ENSG00000287671
NDST3	Lavenderblush3	ENSG00000273275, ENSG00000255595, ENSG00000285582, LY86-AS1, LINC00507, ENSG00000287900, MIR4500HG, ENSG00000285634, ENSG00000229618, LETR1, LINC01885, ENSG00000253452, LINC02346, MAL2-AS1, LINC01331, LINC01476, ENSG00000278727, ENSG00000260412, ENSG00000233928, ENSG00000223944, ENSG00000261037, SLC26A4-AS1, ENSG00000287018, ENSG00000255087, LINC01250, NA, INHBA-AS1, ENSG00000253553, ENSG00000248837, ENSG00000287038, ENSG00000240291, LINC01332, ENSG00000285572, ENSG00000286386, ACAP2-IT1, ENSG00000286286, ENSG00000262267, ATP2B1-AS1, ENSG00000286720, CTXN2-AS1, LINC00488, ENSG00000287439, ENSG00000230393, MIR137HG, THSD4-AS1, CFAP20DC-AS1, ENSG00000256538, ENSG00000249453, ENSG00000258526, LINC00642
DRD5	Brown	COPG2IT1, RFPL1S, FLRT2-AS1, ENSG00000251095, ENSG00000258768, ENSG00000286125, MSC-AS1, ENSG00000257522, MAP4K3-DT, ENSG00000255202, ENSG00000258945, LINC01140, DCTN1-AS1, PART1, ENSG00000281160, ENSG00000260163, ENSG00000272944, CDH13-AS2, LINC02857, ENSG00000231863, TUBA1B-AS1, ENSG00000267396, ENSG00000283183, ENSG00000286353, ENSG00000286282, LINC01963, ENSG00000260920, ENSG00000288062, NA, LINC01182, DPP10-AS1, TSC22D1-AS1, ENSG00000286971, ENSG00000197332, ENSG00000233290, ENSG00000260838, OIP5-AS1, ENSG00000236377, ENSG00000266573, ENSG00000274718, LIN28B-AS1, ENSG00000257434, THCAT155, ENSG00000286342, ENSG00000260966, ENSG00000261167, RAPGEF4-AS1, LUARIS, ENSG00000260108, ENSG00000259985

**Table 6:** Common differentially expressed lncRNAs in hippocampus and fusiform gyrus that are co-expressed with differentially expressed AD-related protein coding genes.

Ensembl ID	DE lncRNA symbol	Co-expressed DE protein coding genes
ENSG00000225465	RFPL1S	GABRA3
ENSG00000274718	–	GABRA3
ENSG00000237737	DCTN1-AS1	GABRA3
ENSG00000271614	ATP2B1-AS1	PLK2; NDST3
ENSG00000226519	LINC00390	PLK2
ENSG00000278727	–	PLK2; NDST3

## 4 Discussion

The etiology of AD is still largely unknown and, except for the rare familiar cases (< 5% of all cases), the disease occurs sporadically with a late onset (Tanzi, 2012). Thus, there is an urgent need to strengthen efforts to understand the pathophysiological mechanisms that lead to AD development.

In this context, the present work aims at contributing to the current knowledge about the pathologic transcriptomic landscape of the human AD brain, as the definition of the changes of gene expression in the AD brain might provide insight to further research in the disease molecular processes. To date, several transcriptome profiling studies have investigated gene expression changes in the AD brain (Annese et al., 2018; Friedman et al., 2018; Srinivasan et al., 2020; Crist et al., 2021; Cain et al., 2023) but a complete set of genes and pathways deregulated in AD is far from established.

By using advanced bioinformatic approaches, we reanalyzed two RNA-seq datasets, one derived from the hippocampus and the other from the fusiform gyrus of AD individuals, matched with healthy controls. We provide here a comprehensive reanalysis of data already published. This is a common and valuable practice in bioinformatics as it optimizes data exploitation considering both new biological knowledge (i.e., new annotated pathways), the updated gene annotation, particularly relevant for lncRNAs addressed in the present study, with most recent advances in bioinformatics approaches. In addition, the combined data analysis from multiple studies (i.e., hippocampus and fusiform gyrus in the present study) can enhance statistical power leading to more reliable identification of deregulated pathways.

Regarding the choice of the selected brain regions, the hippocampus, which is relevant for memory processes, is among the first brain regions that manifest the pathological phenotype of AD. The fusiform gyrus is important for the elaboration of visual stimuli and in particular for facial recognition. While it is known that the hippocampus is a brain area vulnerable in AD (West et al., 2000), little is known about the relationship between the neurodegenerative damage sustained by fusiform gyrus and the pathologic manifestations of the disease. The fusiform gyrus is interested by the neurodegeneration in the subsequent stage of the disease, according to the Braak staging system (Macedo et al., 2023) and a correlation may be found between the inability of patients to recognize familiar faces as the disease progresses and the damage sustained by this region (Ma et al., 2020). In AD, various regions of the brain exhibit the hallmark pathological features associated with the condition that are NFTs and senile plaques. Each of these regions, characterized by their distinct histological and functional properties, appears to be uniquely susceptible to the disease’s progression (Crist et al., 2021). Consequently, the transcriptomic alterations observed in these diverse brain areas may differ, reflecting their individual responses to AD pathology. In this context, the identification of shared molecular changes across brain regions affected in different stages of the disease holds significant importance. Such common alterations could indicate the presence of underlying molecular mechanisms that contribute to the development and progression of AD. Unraveling these shared molecular signatures could provide valuable insights into the fundamental pathological processes driving AD pathogenesis.

The hippocampus RNA-seq dataset derived from patients that were accurately stratified (Annese et al., 2018) and the same criteria were applied to select samples from the original fusiform gyrus RNA-seq dataset (Friedman et al., 2018). Thus, although the two cohorts were not large, the low variability among AD patients and relative controls could contribute to the robustness of our analyses and results. We are aware that analyzing larger datasets would directly improve the statistical power of the approach adopted to give more strength to the results. Although several AD RNA-seq datasets are available, they did not offer the level of stratification we desired or they come with access restrictions as it is the case for the longstanding ROSMAP project (Bennett et al., 2018).

The continuous work in the gene annotation field, by projects as ENCODE and FANTOM (Snyder et al., 2020; Abugessaisa et al., 2021), allowed us to identify more genes in total and more DE genes with respect to the original analyses of the two datasets. Although the two brain regions are underlying different in cytologic terms, the comparative analysis of the two DE gene groups identified 1,082 DE genes in common between the two brain regions, largely with the same deregulation behavior, since only 39 genes showed an opposite deregulation. Thus, these loci may represent a common sign of deregulation, and establish a novel knowledge resource to shed light on the way different areas of the brain are engaged by the AD neurodegenerative process.

In this study we focused our attention on DE lncRNAs, because, as epigenetic regulators of brain functions, their deregulation could be directly involved in AD pathogenesis. The function of the majority of lncRNAs in the brain and their role in the disease is not yet known. So far, the best documented lncRNAs in AD are those involved in AD hallmarks, as antisense transcripts of known AD-related genes, and many more lncRNAs are likely to be operating in trans in neurodegenerative diseases (Riva et al., 2016). We characterized the biotype of the DE lncRNAs identified in the two regions and we found that most of them belong to the antisense category. Thus, the identified DE antisense lncRNAs might alter many and different cellular processes, as it is known that antisense lncRNAs interact with the sense RNA (affecting splicing, polyadenylation, stability, nuclear transport, etc.) but also they act as chromatin modifiers, by establishing complexes with DNA and proteins, such as RNA–DNA duplexes and RNA-protein complexes, that may influence gene transcription (Gagliardi et al., 2018). For example, the Dynactin Subunit 1 (DCTN1) is known to play a critical role in microtubule stability, a biological process increasingly recognized as a potential therapeutic target for tau pathology (Rayaprolu et al., 2021). *DCTN1-AS* is the antisense of *DCTN1* gene and we found this lncRNA downregulated in both brain regions and co-expressed with the same AD-related genes. *DCTN1* was cited as a hub gene within a proteomics-based interaction network module in a study aimed at unraveling the proteopathic biochemical phase of AD (Rayaprolu et al., 2021). We may speculate that *DCTN1-AS* could interact with the *DCTN1* gene or its transcript, potentially modulating its function, within a pathway involving *GABRA3*. Similarly, *STARD4,* a gene regulating the lipid metabolism, possesses an antisense gene. It was found deregulated in AD in a differential expression analysis, comparing *APP/PS1* and healthy murine models with the aim of investigating the process of spine turnover (Heiss et al., 2017). We found *STARD4-AS* as a downregulated lncRNA in AD hippocampus and it could be a valid candidate for investigating its role in the regulation of *STARD4* expression.

To gain insights into the function of the DE lncRNAs, we used the WGCNA bioinformatic tool that is capable of building gene correlation networks and identifying *modules* of co-expressed genes, with the final goal of studying the system-level functionality of genes. LncRNAs that result to be correlated to better-known protein coding genes by similar expression patterns (co-expressed) may be involved in the same cellular functions and molecular pathways. For this reason, gene co-expression networks may help formulating significant predictions about the function of lncRNAs.

Several co-expression and differential co-expression network analyses have already been applied on RNA-seq data from hippocampus and fusiform gyrus, leading to the identification of co-expressed networks and genes associated with AD (Sato et al., 2019; Crist et al., 2021; Xia et al., 2022; Ribeiro-dos-Santos et al., 2023). As our aim was the identification of lncRNAs that could take part of the pathologic molecular mechanisms of AD, we prioritized modules comprising the majority of lncRNAs which could be identified as driver genes and could be postulated to influence the expression of other genes or that could be directly involved in the causal mechanisms of AD. The validity of this approach was confirmed by the pathway enrichment analysis of the prioritized modules for each brain region that highlighted that these modules were related to neurotransmission, memory consolidation and/or neurological diseases (Supplementary Tables S7, S8). Interestingly, we found several DE genes that were present in both hippocampus and fusiform gyrus prioritized modules (Table 3). These targets may result co-regulated or members of the same functional pathway and investigating their function may lead to understanding pathogenetic pathways common to the two brain regions. Several lncRNAs were identified as co-expressed with these DE coding genes related to AD in the prioritized modules for the two brain regions (Tables 4, 5). A limitation of existing co-expression analyses is that they focus on driver genes without offering any insight on their co-expression neighborhood in the modules they have been assigned to. On the contrary, our analysis identified lncRNAs that resulted closely co-expressed with DE coding genes related to AD and, although their co-expression may be casual, the possibility of discovering new interactors of yet unknown pathological mechanisms of AD is worthy of future investigation. As a result of this co-expression analysis, we found six DE lncRNAs (*RFPL1S*, *ENSG00000274718*, *DCTN1-AS1*, *ATP2B1-AS1*, *LINC00390* and *ENSG00000278727*) that are co-expressed with the same AD related coding gene in both hippocampus and fusiform gyrus (Table 6). The function of these lncRNAs is unknown. Two of these co-expressed lncRNAs, as *ENSG00000260163* and *LINC01962* were reported in studies investigating the correlation between the expression of lncRNAs and drug abuse (Bannon et al., 2015; Rompala et al., 2023), while their involvement in AD has not been investigated. *LINC0839* is known to enhance the expression of glioma stem cell lines (Kobayashi et al., 2024). Hence, all these lncRNAs represent a source for further molecular studies aimed at elucidating their function that could shed light on the unknown pathogenic mechanisms of AD.

Finally, having the hippocampal samples from AD patients of the Annese et al. paper (Annese et al., 2018) available, by ddPCR, we analyzed the expression of seven deregulated lncRNAs (*MAP4K3-DT, MEG9, MEG8, PCA3, HAR1A, NECTIN3-AS1* and *STARD4-AS*), chosen as their expression is supported by the GENCODE annotation and four of them (*NECTIN3-AS1, MAP4K3-DT, PCA3,* and *HAR1A*) resulted deregulated also in the AD fusiform gyrus. The deregulated expression of these lncRNAs was confirmed as we observed the same trend of decrease of the RNA-seq analysis (Figure 2), demonstrating the consistency of the RNA-seq bioinformatic analysis and the robustness of the computational approach used to design the primer pairs. Interestingly, the expression of *MAP4K3-DT* has already been found to be altered in AD brains, through a multi-omic data analysis by Klein et al. (2020). *MEG8* is a member of a lncRNA cluster, including *MEG3* and *MEG9*, involved in the response to glycine stimulation in a N-methyl-d-aspartate glutamate receptors (NMDAR)-dependent manner in a murine model (Tan et al., 2017) and this is relevant because the NMDA signaling is impaired in AD (Dore et al., 2017). *MEG9* has been recently reported to be downregulated in AD hippocampus (Wang et al., 2022) and involved in the pathogenesis of autoimmune and neurodegenerative diseases (Plewka and Raczynska, 2022). Regarding *PCA3*, it was extensively studied in cancer (Lemos et al., 2019) and was found to be differentially expressed in the exosomes of cerebrospinal fluid of AD patients (Gui et al., 2015). Finally, in a study focusing on the network of miRNA sponges for various neuropsychiatric disorders, including autism, *HAR1A* was identified as a candidate for this role in the autism spectrum disorder (Balasubramanian and Vinod, 2022) and was also found to be downregulated in AD (Li and De Muynck, 2021).

## 5 Conclusion

Our results demonstrate the existence of specific and common deregulation of the expression profile of the hippocampal region and the fusiform gyrus of AD patients. We are aware that our data require functional investigation of the involvement of deregulated lncRNAs in AD. In fact, correlation analyses are excellent tools for predicting the putative involvement of genes into functional pathways, but they cannot demonstrate it, nor can they provide evidence for causal relationships between a gene and the neurodegenerative process they are correlated with. However, the common deregulated lncRNAs in AD hippocampus and fusiform gyrus of AD patients still offer a valuable shortlist of candidates to be investigated for their involvement in the AD pathogenesis and for the design of novel therapeutic approaches based on lncRNAs.

## Data availability statement

The original contributions presented in the study are included in the article/Supplementary material, further inquiries can be directed to the corresponding author/s.

## Ethics statement

Ethical approval was not required for the studies involving humans because the present work re-analyzed already published datasets. RNA used in the ddPCR analysis was extracted from hippocampal samples obtained from non-profit brain banks, which gathered the consents from patients. The studies were conducted in accordance with the local legislation and institutional requirements. The human samples used in this study were acquired as part of a previous study for which ethical approval was obtained. Written informed consent to participate in this study was not required from the participants or the participants’ legal guardians/next of kin in accordance with the national legislation and the institutional requirements.

## Author contributions

EF: Investigation, Methodology, Writing – original draft. EP: Conceptualization, Supervision, Writing – review & editing. AT: Conceptualization, Supervision, Writing – review & editing. GP: Conceptualization, Supervision, Writing – review & editing. AD'E: Supervision, Writing – review & editing.

## Footnotes

^1^ https://www.bioinformatics.babraham.ac.uk/projects/fastqc/
