# Supplementary material for: Identification of deregulated lncRNAs in Alzheimer’s disease: an integrated gene co-expression network analysis of hippocampus and fusiform gyrus RNA-seq datasets
Source: Front Aging Neurosci. 2024 Jul 17;16:1437278. doi: 10.3389/fnagi.2024.1437278 (PMC11288953; doi:10.3389/fnagi.2024.1437278)
Supplement: Supplementary file 1 [file Table_1.docx]

**Supplementary Table 1. Primer sequences of the lncRNAs chosen for experimental validation by ddPCR.** The gene symbol, the ENSEMBL gene ID, and the primer sequences are reported.

| **Gene Symbol** | **Ensembl ID** | **Primer Forward (5’-3’)** | **Primer Reverse (5’-3’)** |
| --- | --- | --- | --- |
| **MAP4K3-DT** | ENSG00000231312 | TGAAAAGAGGCAGAGTCTTGCT | TCCCTTCTGCTGCCAGATCTT |
| **MEG9** | ENSG00000223403 | TGGACAGGAGGAAGCACACG | GTCTTTTCAGCAGCCTCGCC |
| **HAR1A** | ENSG00000225978 | AGCCAGGGCAAGAGTCTCAG | TCTTCTCTGCCTGGAACGCC |
| **NECTIN3-AS1** | ENSG00000242242 | GGAAAATCTGGAATAGCATCTTC | TGGTGTCCACTGGAAGGCAAG |
| **STARD4-AS1** | ENSG00000246859 | CCAGGCTGCTGCTCAGGTAA | TAACTTCTGGCTTCCTGTTCTC |
| **MEG8** | ENSG00000225746 | GGAGTGTGCAGACCTGTAATGA | AGGTCTCCAGGCTCCATCCT |
| **PCA3** | ENSG00000225937 | GTACAATATCAGAGTTCTGGAAG | CTGTCACGTGAGGACACAGC |
