# Supplementary material for: Identification of deregulated lncRNAs in Alzheimer’s disease: an integrated gene co-expression network analysis of hippocampus and fusiform gyrus RNA-seq datasets
Source: Front Aging Neurosci. 2024 Jul 17;16:1437278. doi: 10.3389/fnagi.2024.1437278 (PMC11288953; doi:10.3389/fnagi.2024.1437278)
Supplement: Supplementary file 2 [file Table_2.docx]

**Supplementary Table 2.** The ddPCR thermal cycle used.

|  | 5 min. | 30 sec. | 1 min. | 5 min. | | 5 min. | | 30 min. | |  |
| --- | --- | --- | --- | --- | --- | --- | --- | --- | --- | --- |
| GAPDH | 1X | 45X | | | 1X | | 1X | | 1X | |
|  | 95 °C | 95°C | 64°C | 4°C | | 90°C | | 4°C | |  |
| MAP4K3-DT | 1X | 45X | | | 1X | | 1X | | 1X | |
|  | 95 °C | 95°C | 64°C | 4°C | | 90°C | | 4°C | |  |
| MEG9 | 1X | 45X | | | 1X | | 1X | | 1X | |
|  | 95 °C | 95°C | 64°C | 4°C | | 90°C | | 4°C | |  |
| HAR1A | 1X | 45X | | | 1X | | 1X | | 1X | |
|  | 95 °C | 95°C | 62°C | 4°C | | 90°C | | 4°C | |  |
| NECTIN3-AS1 | 1X | 45X | | | 1X | | 1X | | 1X | |
|  | 95 °C | 95°C | 62°C | 4°C | | 90°C | | 4°C | |  |
| STARD4-AS1 | 1X | 45X | | | 1X | | 1X | | 1X | |
|  | 95 °C | 95°C | 62°C | 4°C | | 90°C | | 4°C | |  |
| MEG8 | 1X | 45X | | | 1X | | 1X | | 1X | |
|  | 95 °C | 95°C | 64°C | 4°C | | 90°C | | 4°C | |  |
| PCA3 | 1X | 50X | | | 1X | | 1X | | 1X | |
|  | 95 °C | 95°C | 62°C | 4°C | | 90°C | | 4°C | |  |

The ramp rate was set at 2°C/second
