# Supplementary material for: Identification of deregulated lncRNAs in Alzheimer’s disease: an integrated gene co-expression network analysis of hippocampus and fusiform gyrus RNA-seq datasets
Source: Front Aging Neurosci. 2024 Jul 17;16:1437278. doi: 10.3389/fnagi.2024.1437278 (PMC11288953; doi:10.3389/fnagi.2024.1437278)
Supplement: Supplementary file 9 [file Data_Sheet_1.PDF]

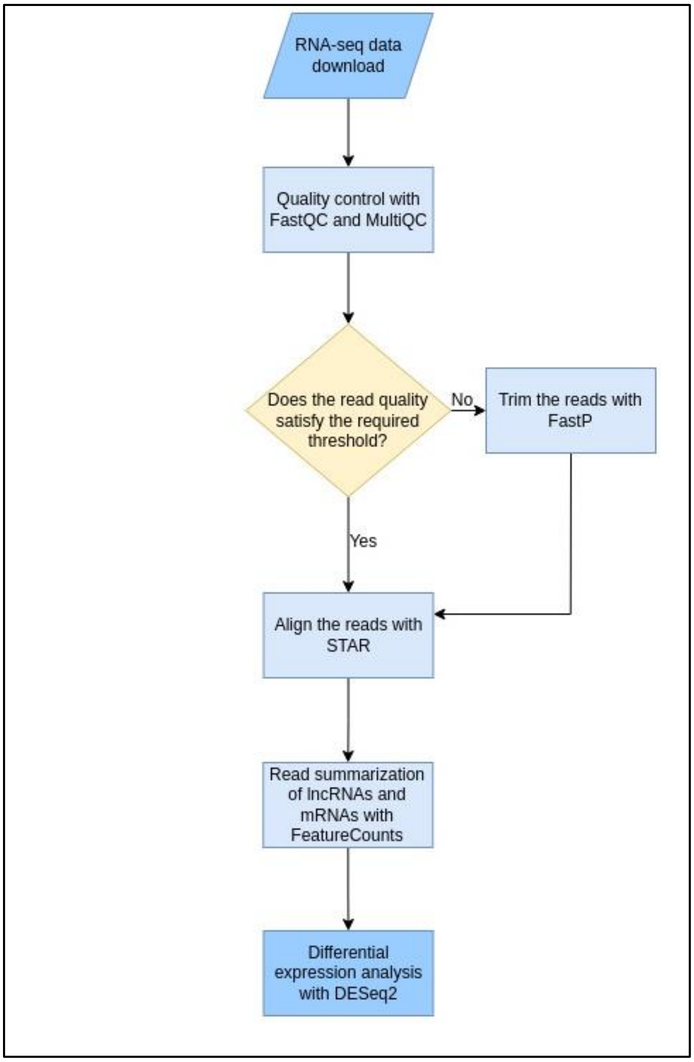


**Supplementary Figure 1.** Workflow of the differential expression analysis employed in this study

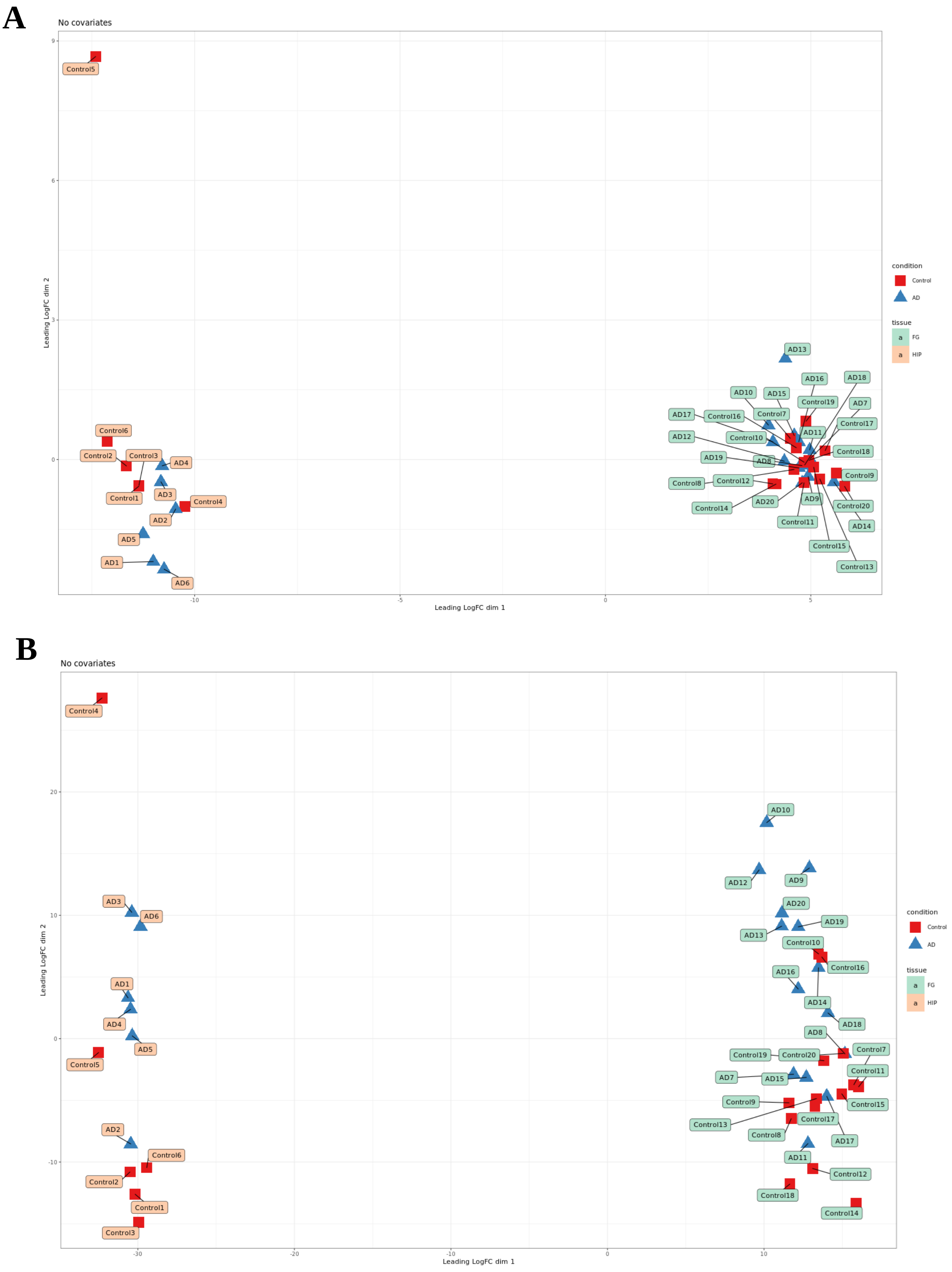


**Supplementary Figure 2.** Multidimensional scaling analysis of **(A)** the rlog transformed gene counts (DESeq2) and **(B)** untransformed normalized gene counts.

A

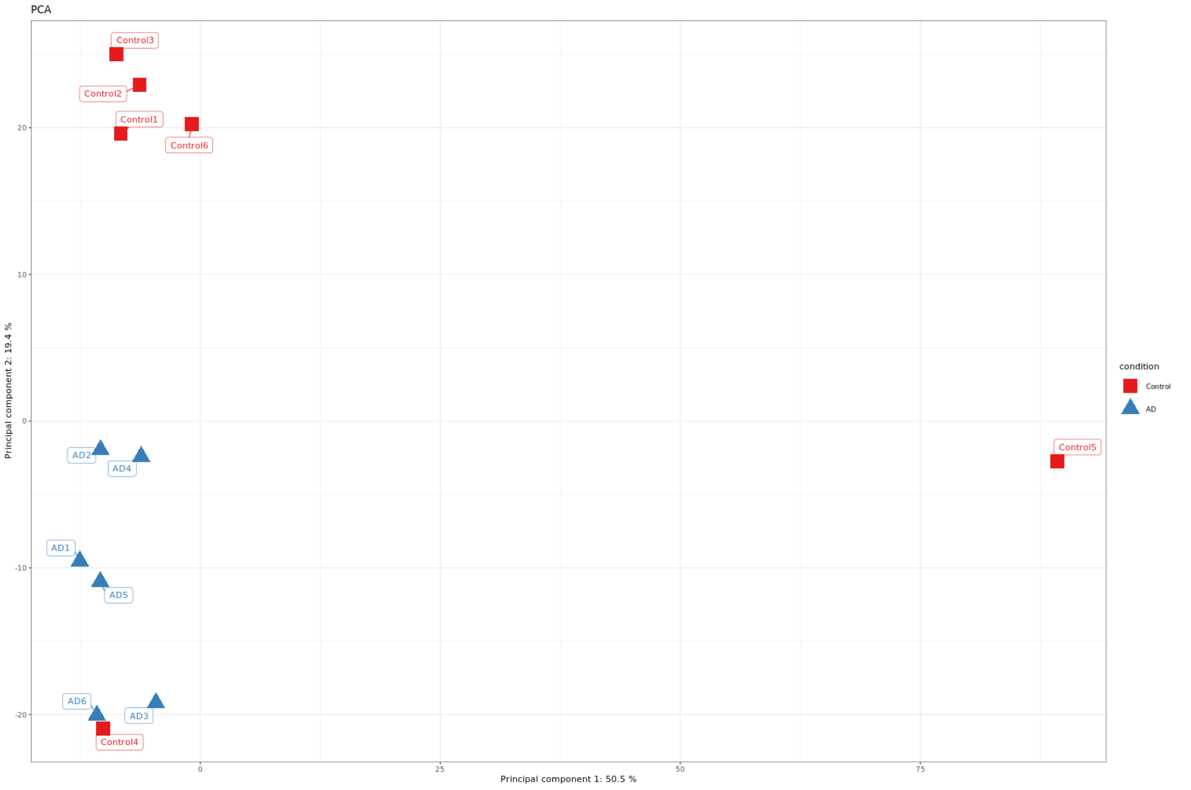


B

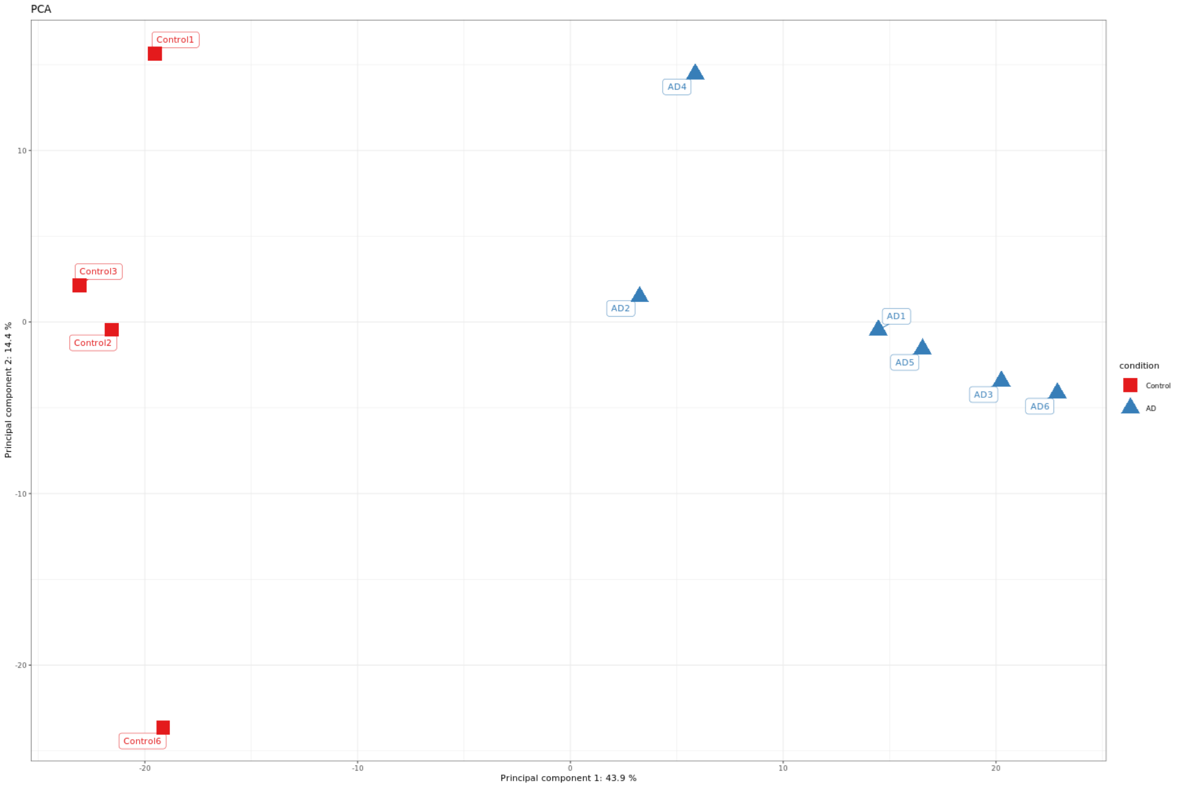


**Supplementary Figure 3.** PCA analysis of the hippocampus RNA-seq samples considered. **(A)** On the basis of their clustering behavior, Control4 and Control5 samples were considered outliers and excluded from downstream analyses. **(B)** Control4 and Control5 removal allowed samples to cluster according to their group.

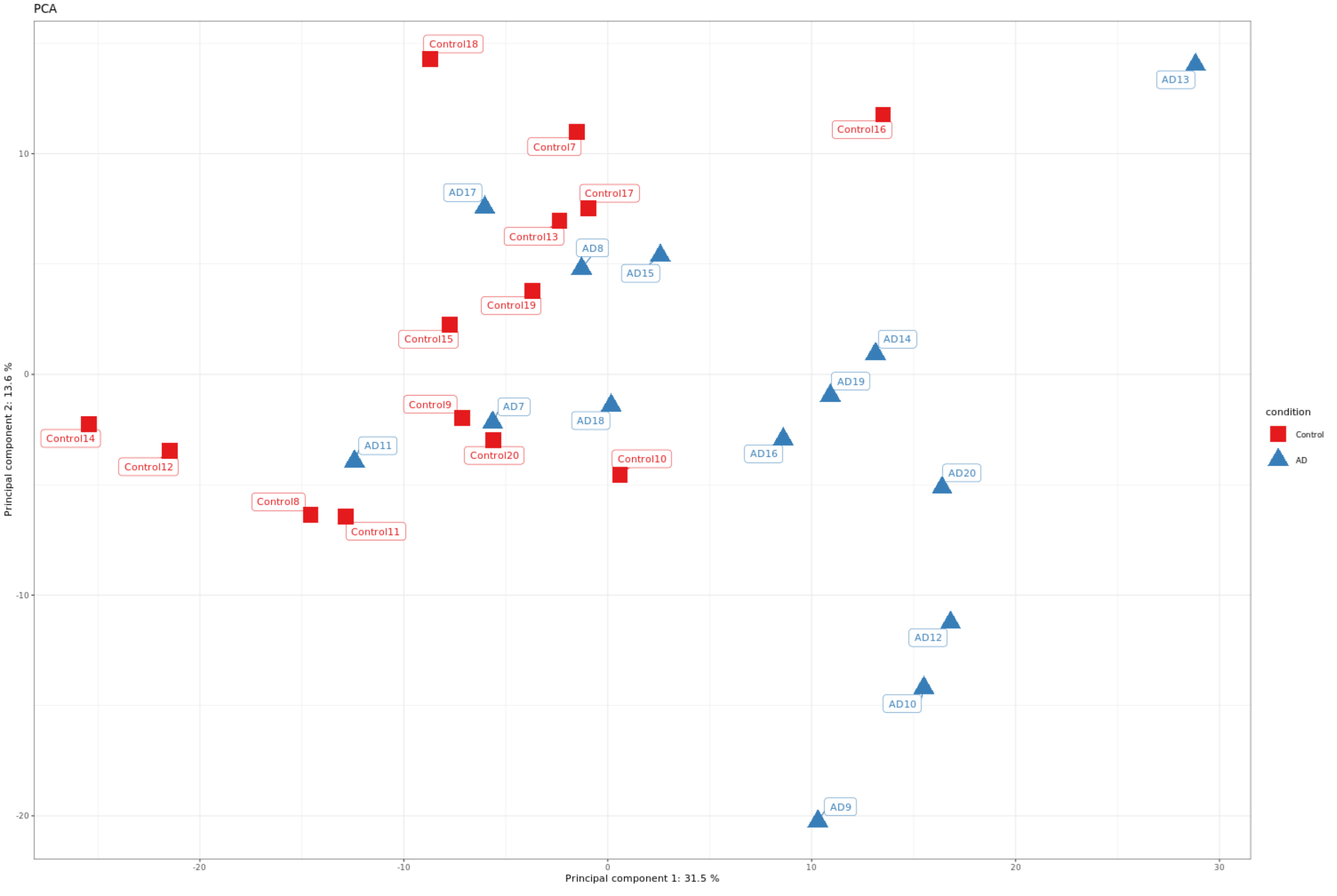


**Supplementary Figure 4.** PCA analysis of fusiform gyrus RNA-seq samples considered.

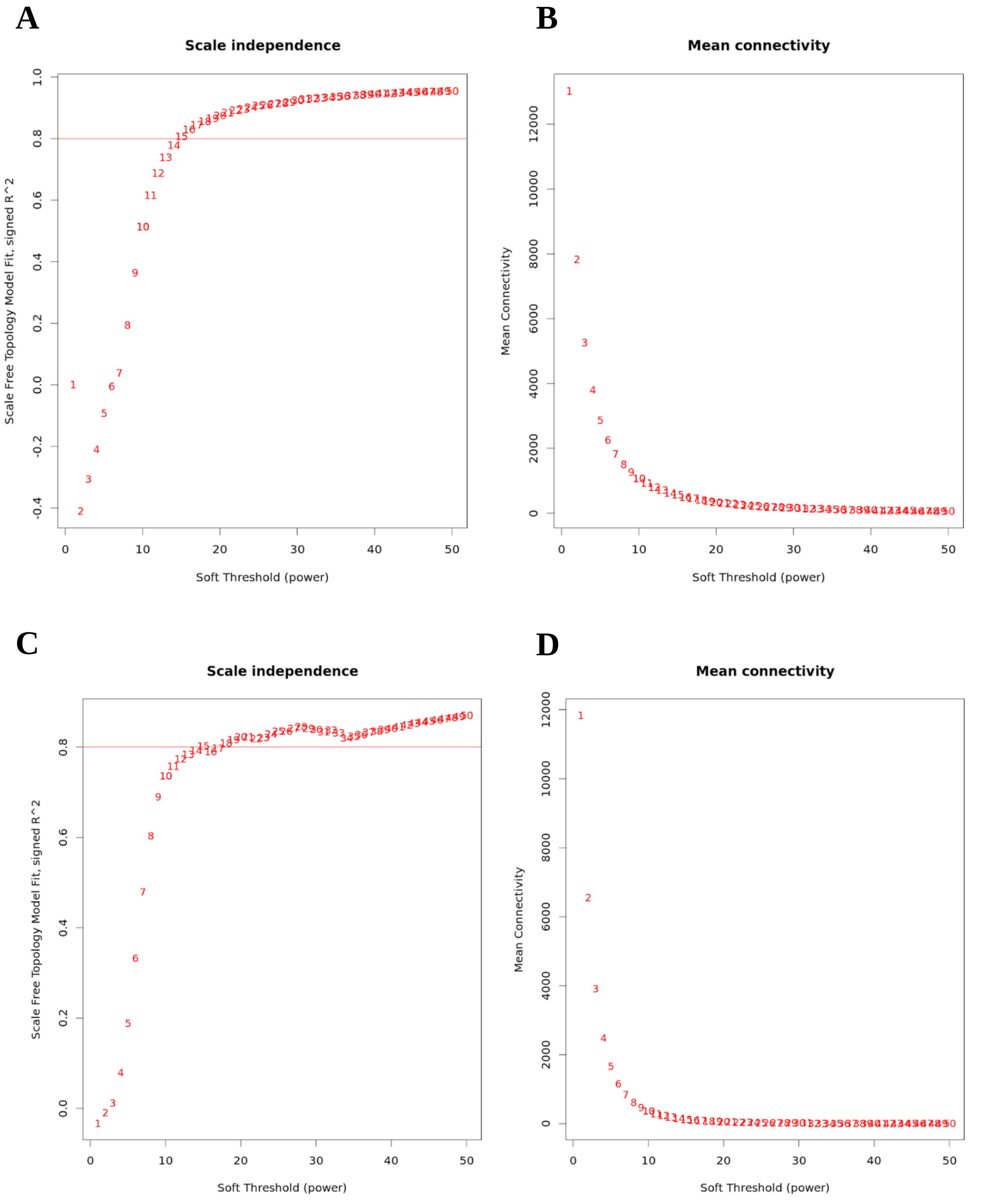


**Supplementary Figure 5.** Plots describing the relationships between soft thresholding powers, the scale free topology model and network mean connectivity. The goodness of fit to the scale free topology model is plotted as signed $R^2$ versus soft thresholding powers for Hippocampus **(A)** and Fusiform gyrus **(C)** datasets. Mean connectivity decreases inversely to powers: for the Hippocampus dataset, $\beta$=15 **(B)** for the Fusiform gyrus dataset, $\beta$=18 **(D)** were chosen as a balanced compromise to highlight correlations, approximate the scale free topology criterion and preserve the mean connectivity of the network at the same time.

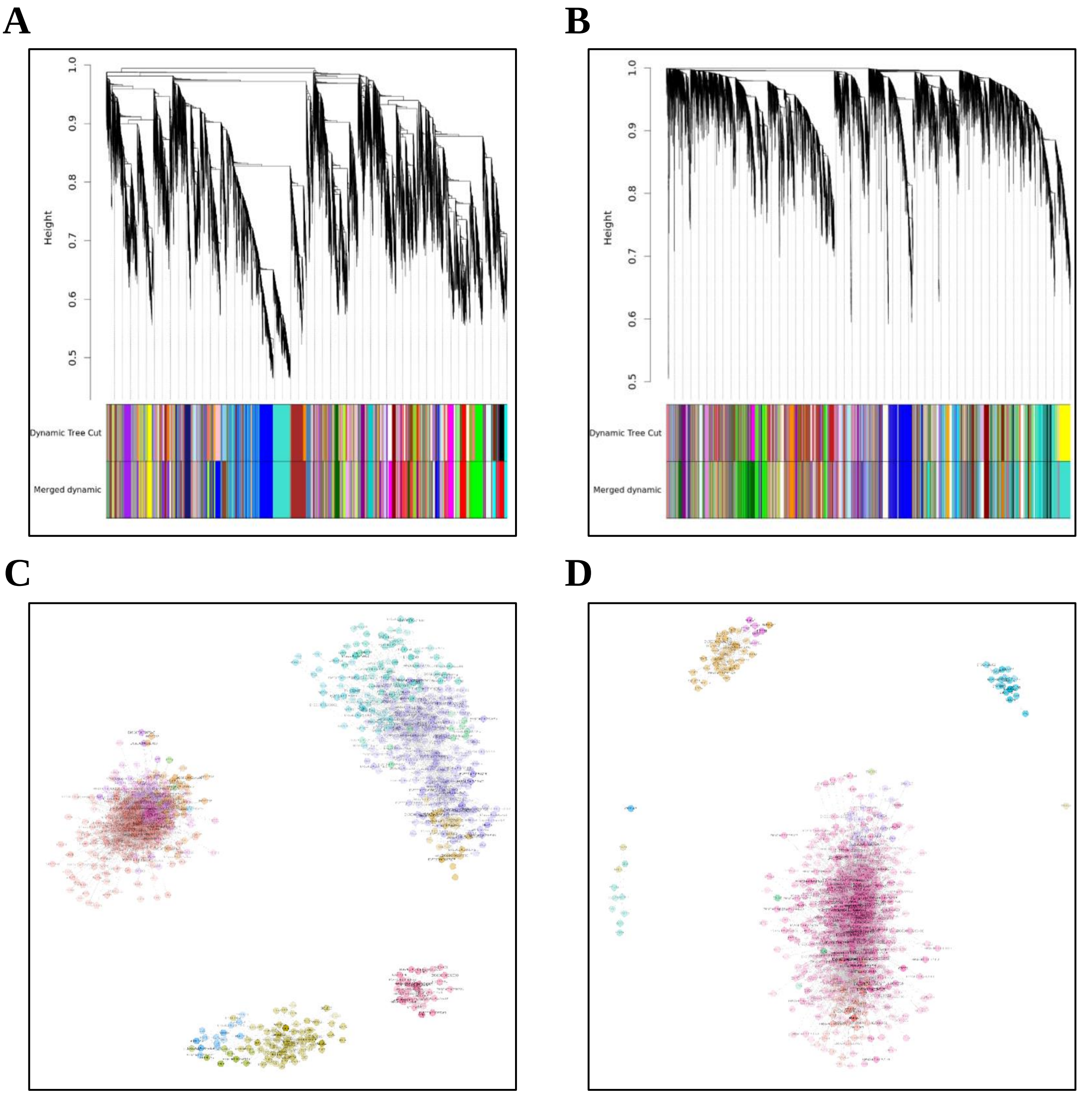


**Supplementary Figure 6:** The result of the correlation analysis performed with WGCNA. **A** and **B**) Dendrogram produced with the hierarchical clustering procedure (A: Hippocampus, B: Fusiform Gyrus). Each leaf harbors a gene and dendrogram branches are clustered into gene 'modules' represented as colored bands below the dendrogram. The final modules obtained after the dynamic merging process are shown as well. **C** and **D**) The gene co-expression network represented with igraph (C: Hippocampus, D: Fusiform Gyrus). The nodes of the network, that are the genes analyzed, are colored according to the WGCNA module they belong to (empirical thresholds were applied and the hippocampus genes of the 'blue' module were not represented for image clarity).
